# The effects of a nutritional packet (live yeast, vitamins C and B1, and electrolytes) offered to steers in a calf-fed system on growth performance, nutrient digestion, feeding behavior, carcass characteristics, and ruminal variables

**DOI:** 10.1093/tas/txad073

**Published:** 2023-07-01

**Authors:** Camron J Rush, Jhones O Sarturi, Nelson O Huerta-Leidenz, Dale R Woerner, Whitney L Crossland, Darren D Henry, Kaliu G S Silva, Alejandra M Lopez

**Affiliations:** Department of Animal and Food Sciences, Texas Tech University, Lubbock, Texas 79409, USA; Department of Animal and Food Sciences, Texas Tech University, Lubbock, Texas 79409, USA; Department of Animal and Food Sciences, Texas Tech University, Lubbock, Texas 79409, USA; Department of Animal and Food Sciences, Texas Tech University, Lubbock, Texas 79409, USA; Department of Animal and Food Sciences, Texas Tech University, Lubbock, Texas 79409, USA; Department of Animal and Dairy Science, University Of Georgia, Tifton, Georgia 31793, USA; Department of Animal and Food Sciences, Texas Tech University, Lubbock, Texas 79409, USA; Department of Animal and Food Sciences, Texas Tech University, Lubbock, Texas 79409, USA

**Keywords:** calf-fed, digestibility, electrolytes, live yeast, vitamin B1, vitamin C

## Abstract

Effects of a nutritional packet strategically offered to calf-fed system steers on growth performance, nutrient digestibility, feeding behavior, ruminal variables, and carcass characteristics were evaluated. Angus crossbred steer-calves (*N* = 60; body weight [BW] = 234 ± 4 kg) were used in a randomized complete block design (block = BW) and stratified into two treatments: 1) control; and 2) 30 g/steer-daily (dry matter [DM] basis) of a nutritional packet containing (steer-daily basis): Live yeast (*Saccharomyces cerevisiae*; 1.7 × 10^10^ CFU), vitamin C (Ascorbic acid, 162 mg), vitamin B1 (thiamin hydrochloride, 400 mg), sodium chloride (2.4 g), and potassium chloride (2.4 g). Animals were offered (electronic feed-bunks [SmartFeed, C-Lock Inc., Rapid City, SD]), a steam-flaked corn-based finishing diet to ad libitum (individual intake), once daily for 233 d. Treatments were offered during the first and last 60 days on feed (DOF). The GLIMMIX procedure of SAS was used, with steer as the experimental unit, treatment and phase (for feeding behavior and digestibility) as fixed effects, and BW-block as a random effect. Steers offered the nutritional packet had 14% less (*P* < 0.01) intake and 18% greater (*P* = 0.01) feed efficiency during the initial 30 DOF. Intake (days 0 to 233) was 6% greater (*P* = 0.02) for steers offered the nutritional packet, while BW gain was not different (*P* ≥ 0.44). Greater (*P* = 0.02) dressing percent (61.1% vs. 62%) for steers offered the packet was observed, while other carcass variables were not different (*P* ≥ 0.33). Digestibility of DM, organic matter, and fiber were greater (*P* < 0.01) for steers offered the packet. Steers offered the packet spent 13% less time eating during the first 60 DOF, while during the last 60 DOF a 14% greater meal frequency and 12.3% smaller mean meal size (treatment × phase interaction, *P* < 0.02) were observed. Steers offered the packet had a reduced (*P* ≤ 0.01) mean meal duration during both phases. Regardless of treatment, a decreased rumination (*P* ≤ 0.03) and chewing (*P* ≤ 0.01) activities were observed for the last 60 DOF compared to the first 60 DOF. Ruminal papillae area was 30% greater (*P* = 0.02) and the total volatile fatty acid (VFA) tended (*P* = 0.09) to be greater for steers offered the nutritional packet. The nutritional packet offered to calf-fed steers improved feed efficiency during the initial 30 d after arrival, while inducing superior overall intake, nutrient digestibility, dressing percentage, ruminal papillae area, and total ruminal VFA.

## INTRODUCTION

Newly weaned calves entering a calf-fed feedlot system are vulnerable to subacute digestive challenges, which may encumber a reduction in feed intake that can last for several weeks (Fluharty et al., 1994). Furthermore, parameters such as ruminal volume, dry matter (DM) content, and the number of protozoa may also decline as the fasting period prolongs (Fluharty et al., 1995). An elevated gastro-intestinal load of parasites (Dahmer et al., 2022) may also aggravate such a reduction in feed intake. Feeding high grain diets earlier in the life of terminal cattle has the advantage of greater feed efficiency and fewer overall days on feed (DOF; López-Campos et al., 2013). Therefore, optimizing the calf-fed system is of great importance in the context of sustainable beef production.

Stressors such as environmental and dietary changes, transportation, and handling can alter the nutritional status of calves and disrupt homeostasis (Grandin, 1997). Dehydration, low energy, and depletion of anti-oxidants at feedlot entry not only could hinder gain performance but also threaten immune status as well (Owens et al., 1998). Nonantibiotic and nutritionally justified strategies that can offset the negative effects of the movement of calves into the feedlot are strongly encouraged.

Research has indicated that nutritional technologies may positively affect calves entering the feedlot. For instance, strategies involving the dietary inclusion of live yeast, vitamin C (ascorbic acid), vitamin B1 (thiamin), and electrolytes (generally not used in combination), have shown individual evidence of positive effects on the host, which may elicit a potential positive associative effect when perhaps offered within a single dietary mixture.

Live yeast stimulus in ruminal cellulolytic bacterial growth and consequent positive effects on fiber digestion have been reported in beef cattle (Ogunade et al., 2019). It has been suggested that vitamin C accumulates within activated neutrophils, and depending on current physiological conditions, an increased recycling and use of external vitamin C may be enhanced (Washko et al., 1993). In addition, the supplementation of thiamin improved the expression of thiamin transporters in the rumen epithelium of dairy cattle, and attenuated the inflammatory process induced by high-grain diets offered to low-production dairy cows (Pan et al., 2017). Both live yeast and vitamin B1 (although not offered in combination) have been suggested to improve ruminal conditions due to an improvement on the relative abundance of ruminal lactic acid utilizing bacteria and increased ruminal pH average in beef (Ogunade et al., 2019) and dairy cattle (Wang et al., 2014; Pan et al., 2016). Schaefer et al. (1992) reported a reduction in live-weight loss and improved normalization of blood lactate levels of cattle offered a solution of Na, K, and Mg prior to and following transit. In addition, Schaefer et al. (1997) showed that similar electrolyte therapy used for beef cattle under stressful conditions (management and/or after transportation) induced improvements on hot carcass yield.

The combination of live yeast, vitamin C, vitamin B1, and electrolytes offered to beef long-yearlings on the final portion of the feedlot phase showed improvements on nutrient digestibility, subcutaneous fat deposition, and a more consistent feeding behavior pattern of animals offered a steam-flaked corn-based finishing diet (Nardi et al., 2022). Long-yearlings offered energy-dense finishing diets will be physiologically exposed to additional oxidative stress due to the more prominent rate of adipose tissue deposition occurring in such phase, elevated intake of nutrients and overall body weight (**BW**; Bruns et al., 2004), as well as a potential for an increase in digestive disorders towards the last DOF (Castillo-Lopez et al., 2014). However, recently weaned calves may also be exposed to ruminal challenges due to changes in management, environment, and dietary conditions (Fluharty et al. 1994, 1995). Thus, such a combination of nutritional strategies may also be beneficial as supportive care of newly received calves in feedlots.

Therefore, it was hypothesized that supplement technologies (live yeast, vitamin C, vitamin B1, and electrolytes) combined in a composite nutritional packet would support calf-fed steers with overcoming challenges during the initial days post feedlot arrival and those that precede cattle harvest. The objective of this experiment was to evaluate the effects of a nutritional packet containing *Saccharomyces cerevisiae*, vitamin C, vitamin B1, and electrolytes on the growth performance, feeding behavior, nutrient digestibility, carcass characteristics, papillae morphology, and ruminal volatile fatty acid (**VFA**) profile of steers undergoing a calf-fed system.

## MATERIALS AND METHODS

Experimental procedures in this project with the involvement of animals were performed in accordance with the Texas Tech University Animal Care and Use Committee Protocol 20066-08.

### Receiving Phase and Experimental Design

On December 14, 2020 (day 0), a total of 70 Angus crossbred steers were delivered to the Texas Tech University Beef Center (Idalou, TX). Animals were acquired from a single ranch source located in New Mexico, USA and were transported for approximately 6 h, overnight, until arrival at the research station early in the morning. Calves were not offered creep-feeding strategies but received distiller’s grains cubes (0.23 kg/animal-daily, as-is basis) for approximately 30 to 40 d postweaning (7 to 8 mo of age). Approximately 30 d before shipment, calves received immunization with the same vaccination protocol further described. Upon arrival, BW was recorded on two consecutive-days (days 0 and 1), and animals were processed with the following protocol: Mycoplasma Bovis Bacterin (Myco-B one dose; American Animal Health, Inc. Grand Prairie, TX); BoviShield IBR/BVD Gold (Zoetis, Florham Park, NJ); UltraChoice 7 (Zoetis, Parsipanny-Troy Hills, NJ); and fenbendazole (10%) at 5mg/kg steer BW (Safe-Guard, Merck Animal Health, Madison, NJ). In addition, each steer received a numbered ear tag in the right ear, and a (low frequency) radio frequency identification (**RFID**) tag (Allflex USA, Inc., Dallas, TX) in the left ear for identification purposes and measurement of individual intake. Within the cattle group, 10 animals were not used due to bad temperament or deemed as outside the BW range of the lot. Using the average BW (234 ± 4 kg) collected from days 0 and 1, steers (*N* = 60) were randomly allocated within BW blocks (*N* = 30) and randomly assigned to one of two treatments (*N* = 30 individually fed steers per treatment), following a randomized complete block design. Diets were offered using electronic automated feed intake recording bunk systems (*N* = 8; SmartFeed bunks, C-Lock Inc., Rapid City, SD) and individual feed intake was measured for 233 d. On day 30 of the experiment, each steer was implanted with 100 mg of trenbolone acetate and 14 mg of estradiol benzoate (Synovex Choice, Zoetis) administered by subcutaneous implantation in the middle one-third of the ear, and on day 119, steers were re-implanted with 200 mg of trenbolone acetate and 28 mg of estradiol benzoate (Synovex Plus, Zoetis).

### Diets, Treatments, and Feeding

Throughout the entirety of the experiment, steers were offered diets to ad libitum intake (at least 5% refusal rate) once daily at 1400 h. Upon arrival, steers were offered a diet containing 22% steam-flaked corn and stepped up (every 7 d over the course of 28 d; Table 1) to the final finisher diet containing 65% steam-flaked corn. Steers assigned to the control group were offered 30 g/animal-daily [DM basis] of finely-ground corn (carrier only), while steers assigned to the “nutritional packet” group received the treatment at 30 g/animal-daily (DM basis) containing: 1.7 × 10^10^ CFU/steer-daily of *S. cerevisiae*, 162 mg/steer-daily of ascorbic acid (vitamin C), 400 mg/steer-daily of thiamin hydrochloride (vitamin B1), 2.4 g/steer-daily of sodium chloride (NaCl), and 2.4 g/steer-daily of potassium chloride (KCl) provided by Vigaly, LLC (San Antonio, TX). The Texas Tech University School of Veterinary Medicine Laboratory provided monthly reports of *S. cerevisiae* concentration (5.66 × 10^8^ CFU/g of DM) in the nutritional packet, by following the Food and Drug Administration’s method “Bacterial Analytical Manual”. Briefly, approximately (10 g, as-is) of the nutritional packet was homogenized in a stomacher for 2 min (Seward Ltd, Worthing, UK) with 90 ml of 0.1% buffered peptone water, and serial-diluted until 1 × 10^−7^. Using sterilized instruments, the diluted solution (0.1 mL) was placed on solidified Dichloran Rose Bengal chloramphenicol agar plates using a pipet and spread out on the plate using a bent glass rod. Plates were incubated (25 ºC) in the dark without stacking or inverting and maintained undisturbed and colony-forming units were counted 4 d later.

**Table 1.** Dietary ingredients and nutritional composition offered to calf-fed steers during the adaptation and finisher phase

Item	Step 1	Step 2	Step 3	Step 4	Finisher
Inclusion, DM % basis					
Steam-flaked corn	22	32.75	43.5	54.25	65
Wet corn gluten feed	54.1	45.67	37.25	28.62	20
Low-quality alfalfa hay	20	17	14	11	8
Yellow grease	0	0.75	1.5	2.25	3
Mineral vitamin supplement^1^	2.5	2.38	2.25	2.13	2
Limestone	1.4	1.45	1.5	1.55	1.6
Urea	0	0	0	0.2	0.4
Treatment, g/d^2^	30	30	30	30	30
Analyzed nutritional composition					
Crude protein, %^3^	18.68	17	15.31	14.14	12.97
Acid detergent fiber, %	10.85	10.84	8.9	7.85	5.96
Neutral detergent fiber, %	27.1	27.06	22.96	21.22	17.33
Crude fat, %	2.5	3.2	4.8	5.5	5.67
NEm, Mcal/kg^3^	1.75	1.86	1.95	2.02	2.12
NEg, Mcal/kg^3^	1.13	1.22	1.3	1.37	1.45
Ca, %	1.04	0.88	0.87	1.01	0.9
P, %	0.67	0.63	0.57	0.52	0.47
Mg, %	0.35	0.33	0.32	0.27	0.24
K, %	1.53	1.58	1.55	1.27	1
S, %	0.36	0.33	0.28	0.25	0.2
Na, %	0.34	0.3	0.25	0.21	0.19
Cl, %	0.6	0.6	0.54	0.53	0.42
DCAD	14.31	15.77	17.94	11.15	9.38

^1^Supplement contained (DM basis) 67.7538% carrier (cottonseed meal), 0.5% anti-oxidant (Endox; Kemin Industries, Inc., Des Moines, IA), 3.76% urea, 10% potassium chloride, 15% sodium chloride, 0.0022% cobalt carbonate, 0.1965% copper sulfate, 0.0833% iron sulfate, 0.0031% ethylenediamine dihydroiodide, 0.167% manganous oxide, 0.125% of a 0.2% selenium premix, 0.9859% zinc sulfate, 0.0099% vitamin A (1,000,000 IU/g), and 0.157% vitamin E (500IU/g) and provided (dietary) 30 mg/kg of monensin (0.75% Rumensin-90 in the supplement; Elanco Animal Health, Indianapolis, IN) and 9 mg/kg of tylosin (0.5063% Tylan-40 in the supplement; Elanco Animal Health).

^2^Finely-ground corn for the control steers or nutritional packet (30 g DM per steer-daily).

^3^Calculated using the 2016 Beef Cattle Nutrient Requirement Model (BCNRM).

Feed refusals were removed from each SmartFeed bunk approximately 30 min prior to feeding, aiming to guarantee that electronic bunks always had feed inside, and without any spoilage. Diets for each treatment were mixed fresh every day by using a Roto-Mix trailer (model 84-8, Dodge City, Kansas) for 10 min. Dietary treatments were top-dressed directly inside the mixer during the mixing procedure and the control group was offered diet first to avoid any potential cross-contamination. To avoid any potential next-day feeding cross-contamination, the mixer was subsequently flushed by feeding spare steers (*N* = 10) the control diet.

The total diet amount for each treatment was evenly distributed SmartFeed bunks of each treatment. The assigned treatments were offered for the initial 60 d after arrival (for BW measurements plus additional 3 d of digestibility assessment) and the final 60 d prior to harvest, which defined the phases 1 and 2, respectively. Between phases, there was an intermittent period where treatments were not offered. The DM content of diets and individual ingredients was determined on a weekly basis (in duplicate) using a forced-air oven (Grieve, Round Lake, IL) set at 100 °C for 24 h. Diet samples from each treatment were collected weekly at feeding time, composited (by weight period), dehydrated (55 °C, 72 h in a forced-air oven, Grieve), and ground to 1 mm using a Wiley Mill (Thomas Scientific, Swedesboro, NJ). Dry-ground samples were used for the overall nutritional composition of diets, which was conducted at a commercial laboratory (ServiTech Laboratories, Amarillo, TX) for crude fat, calcium, magnesium, phosphorous, potassium, sulfur, sodium, chlorine, and dietary cation–anion difference (**DCAD**), while acid detergent fiber (**ADF**) and neutral detergent fiber (**NDF**) were measured in house at the Ruminant Nutrition Laboratory (Texas Tech University).

### Body Weight, Intake, and Carcass Measurements

Individual unshrunk BWs were recorded for two-consecutive days on 0, 16, 30, 60, 88, 119, 147, 173, 204, and 233, between 0600 and 1200 h using the Cow Power 1050 Squeeze Chute (Arrow CattleQUip, Manitoba, Canada) equipped with four Tru-Test HD5T load cells (Tru-Test Ltd, Auckland, New Zealand), which have a ± 0.50 kg readability and a Tru-Test ID5000 indicator. Average daily gain (**ADG**) was calculated for each weight period by subtracting the initial BW from the final BW and dividing such value by the DOF for the respective period.

Daily DM intake (**DMI**) of each steer was recorded using SmartFeed bunks, which was adjusted to the weekly dietary DM content assessments. Individual feed consumption data was checked twice daily (morning and afternoon), and for situations in which data was not present (i.e., RFID malfunction or lost), the data was considered missing, rather than numerically zero. The BW feed efficiency (**G:F**) was calculated by dividing the ADG by the DMI from each respective time-period.

On day 233 of the experiment, all steers were shipped to a federally inspected slaughter facility located in Friona, TX (172 km from Idalou, TX). The hot carcass weight (**HCW**), 12^th^ rib fat, longissimus muscle (**LM**) area, marbling score, yield grade, quality grade, and liver score were recorded by coordinating with Texas Tech University trained personnel. Livers were visually assessed for the presence of abscesses, and assigned one of the following grades: 0, no abscesses or scars; A^−^, one or two small abscesses; A, one or two large abscesses or multiple small abscesses; or A^+^, multiple large abscesses. Due to the limited amount of condemned livers, scored liver data were combined and presented as “percent abscessed livers”. Dressing percentage (**DP**) was calculated by dividing the HCW by the respective individual final (day 233) unshrunk BW. The carcass-adjusted final BW was calculated by dividing an animal’s individual HCW by the average dressing percentage of the experiment (61.56%), followed by a 4% pencil shrink adjustment. The carcass adjusted ADG and G:F were calculated using the carcass-adjusted final BW.

### Apparent Total Tract Nutrient Digestibility

Two separate digestibility assessments were conducted for phases 1 and 2. The phase 1 digestibility measurements occurred on days 60 to 63, while the phase 2 digestibility measurements occurred from days 219 to 222. The first phase BW measurements accounted for 60 d only, while treatments were offered for additional 3 d to make sure treatments were included during the digestibility assessment. Fecal samples were collected from each steer in a squeeze chute at 0700 and 1700 h for four consecutive days (*N* = 8 samples per steer) and frozen (–20 ºC) for further analyses. Diet samples from both treatments were collected at feeding time (1400 h) beginning the day prior to the first fecal collection and frozen (–20 ºC) for further analyses. Upon completion of the collection period, fecal samples were dried (55 ºC for 120 h) in a forced air oven, ground (1 mm) using a Wiley Mill, and composited by individual steer per phase. Diet samples were collected daily, composited by treatment, dehydrated at 55 ºC for 72 h, and ground to pass a 1 mm sieve for further laboratory analyses.

Indigestible neutral detergent fiber (**iNDF**) was used as an internal marker to estimate individual total fecal output, as described by Gregorini et al. (2008), Cole et al. (2011), and Krizsan and Huhtanen (2013). Briefly, 0.5 g of diet and fecal sample were weighed in duplicate within Ankom F-57 filter bags and placed inside the rumen of a beef steer (surgically prepared with a ruminal-cannula) for 288 h (steer adapted to a hay-based diet). Bags were removed from the rumen, rinsed with tap water until clear, rinsed with de-ionized-water, and analyzed for NDF content as described by Van Soest et al., 1991. For this process, alpha-amylase, sodium sulfite, and a final rinse of acetone were used, and not corrected for residual ash content. The following equation was used for apparent total nutrient digestibility calculations:


$$\begin{array}{l} Apparent\;nutrient\;digestibility,\;\%\;=\;100-100\;x\\ \left[\frac{conc.of\;iNDF\;in\;feed}{conc.\;of\;iNDF\;in\;feces}\times \frac{conc.\;of\;nutrient\;in\;feces}{conc.\;of\;nutrient\;in\;feed}\;\right] \end{array}$$

### Feeding Behavior (Accelerometer)

Nine steers per treatment were randomly selected to have an ear-tag accelerometer mounted on the RFID (CowManager Sensor, Agis Automastisering BV, Harmelen, the Netherlands) for the entire study period. Validated ear movements were identified through the tag’s sensors’ capability to identify animals’ eating, ruminating, resting, and active times through an algorithm (Bikker et al., 2014; Thomas et al., 2021). Wi-Fi connected antennae were mounted on site between the steers’ location and the office with a computer, which contained the CowManager software. Data from the ear tags were collected continuously. The data was then compiled by phase (1 and 2) in which steers received the treatments in minutes spent on each activity per day. Chewing activity was accounted by adding eating time with rumination time.

### Feeding Behavior Based on Meal Criterion Calculation

Through the utilization of the SmartFeed bunk system, feeding behavior was measured for all steers enrolled in the experiment. Feeding behavior was analyzed individually by phase (1 and 2) by using the Meal Criterion Calculation (MCC; v. 1.9.7919.35834) software, for the following feeding behavior traits: Daily meal frequency, time spent eating, mean meal duration, and mean meal size. Meals within bunk visits were calculated with R software (R Core. Team, 2017) as described by Mendes et al. (2011) and Bailey et al. (2012).

### Ruminal Morphology and Volatile Fatty Acid Profile

Upon cattle harvest at the federally inspected facility, trained TTU personnel collected ruminal fluid and ruminal tissue samples from the cranial sac (*Atrium ruminis*) epithelium. Briefly, immediately after USDA official personnel performed a viscera inspection, the rumen cranial sac was anatomically located, and a ruminal fragment was collected (3 to 5 cm^2^) and placed inside a prelabeled whirl-pak bag. Thereafter, a ruminal compartment opening was performed by a cranial-caudal incision on the left side of the ruminal ventral sac. At that moment, two small-size orifice metal sieves were used to take approximately 2 to 3 L of ruminal content and squeeze approximately 50 mL of liquid inside a 50 mL tube (polyethylene, Falcon conical). Most of the rumen liquid inside the sieves was disposed off prior to collecting the sample, aiming to flush any residual liquid or content from previous specimen collected. The ruminal fluid collected was acidified with 0.5 mL (1% of the volume collected) of a 20% H_2_SO_4_ solution to stop fermentation and preserve volatile compounds (Schulmeister et al., 2020). Collections were performed in approximately 10 to 15 s per animal. After collections, the ruminal fluid tubes were rinsed with tap water and immediately frozen using dry ice, transported to the Ruminant Nutrition Lab (Texas Tech University), and stored at –20 ºC until further analyses. Ruminal tissue samples were rinsed with de-ionized water and submerged in 70% isopropyl alcohol (250 mL/sample) followed by storage under refrigeration (5 ºC) until further morphological analyses. Individual identification of viscera was possible through recording the animal identification slaughter order adjusted to account for condemned carcasses or condemned viscera.

The ruminal tissue fragment processing followed the technique described by Silva et al. (2018), in which tissue fragments were first trimmed with a scalpel to approximately 1 cm^2^, and the papillae in such fragment were counted by three trained individuals to generate an average papillae number per fragment. Following, 12 papillae (representative of the fragment) were randomly removed and placed on a petri dish along with the base of the tissue fragment for scanning procedure. A scanned picture of the petri dish with the samples were then uploaded to the ImageJ (version 1.46r) software to determine the area of tissues. Calculations for each variable were performed as follows:

1) Average papillae number (**APN**), *N*/cm^2^ = average of the three counts of ruminal papillae in the fragment/area of the fragment-base
2) Average papillae area (**APA**), cm^2^ = average area of the 12 ruminal papillae scanned
3) Absorptive surface area (**ASA**), cm^2^/fragment-cm^2^ = 1 + (APN × APA) – (APN × 0.002); where “1” represents the adjusted 1 cm^2^ of the fragment-base, and “0.002” represents the estimated fragment-base area (cm^2^) that each papillae uses when attached to the fragment-base.
4) Papillae absorptive area, % of ASA = [(APA × 2 × APN)/ ASA] × 100; where “2” represents both sides of the papillae area scanned.

Frozen ruminal fluid was thawed overnight (5 ºC) and used to determine VFA total concentration and respective molar proportions according to Vanzant and Cochran (1994). Briefly, centrifugation (10,000 × *g*; 10 min; 4 ºC) was performed, and 4 mL of the supernatant were deproteinized using 0.8 mL of 25% metaphosphoric. Subsequently, VFA molar proportions were determined using gas chromatography (Model 5890, Hewlett-Packard, Avondale, PA) with a flame ionization detector. The chromatograph was fitted with a 1.8-m, 4-mm i.d. glass column, packed with 10% SP1200/1% H3P04 on 80/100 WAW Chromosorb (Supelco, Bellefonte, PA). The column was maintained at 130 °C, the detector and injector were maintained at 250 °C, and carrier gas (N_2_) flow was 60 mL/min.

### Morbidity and Mortality Information Disclosure

In the current assessment, 14 control steers were treated for respiratory infection compared to 7 steers consuming the nutritional packet and 2 control steers died after treatment, in which cause of death was deemed pneumonia determined by necropsy and lung tissue sample assessment by Texas A&M Veterinary Medical Diagnostic Laboratory (Amarillo, TX). One steer from each treatment group was removed from the assessment due to physical injuries nonrelated to treatments.

### Statistical Analysis

Data were analyzed using the GLIMMIX procedure of SAS (SAS Inst., Inc., Cary, NC) with steer being considered the experimental unit. The model included the fixed effect of treatment and the random effect of BW block. Bias of degrees of freedom was adjusted using the Kenward–Roger method. Carcass data (quality grade and liver abscess) were fitted in the same model previously described, although because of the nature of such binomial distribution, the link function of the GLIMMIX procedure of SAS was used for data analyses and treatment effects, and the inverse-link function was used for reporting of responses. Given that the digestibility and the feeding behavioral assessments were performed during the phases 1 and 2 only, phase was used as a repeated measure, in which the fixed effects of treatment, phase, and the interaction treatment × phase were evaluated, and BW block was considered as a random effect. Covariance structures for repeated measures were chosen based on the smallest Akaike Information Criterion. *P*-values of *P* ≤ 0.05 were considered significant and tendencies determined at *P* > 0.05 and ≤ 0.10.

## RESULTS

By design, the initial BW of the experimental groups were not different (*P* = 0.37), with the average BW of 234 ± 4 kg (Table 2). After the first 60 d, or the end of phase 1, there was a tendency (*P* = 0.08) for the control animals to have an increased BW (338 vs. 330 kg; SEM = 5.9). At the conclusion of the experiment neither unshrunk nor carcass adjusted final BW were affected (*P* ≥ 0.53) by treatments (Table 2). During the initial 30 d on feed, steers offered the nutritional packet had 14% less (*P* < 0.01) DMI and 18% greater (*P* = 0.01) feed efficiency. During the intermittent period between phases 1 and 2, when treatments were not offered, animals previously offered the nutritional packet had a 7.7% increase (*P* = 0.04) in ADG compared to the control group. Overall intake (d 0 to 233; Table 2) was 6% greater (*P* = 0.02) for steers offered the packet, while carcass adjusted ADG (1.61 vs. 1.56) and carcass-adjusted feed efficiency (0.198 vs. 0.204) were not affected (*P* ≥ 0.44) by treatments.

**Table 2.** Effects of a nutritional packet on growth performance of calf-fed steers offered a steam-flaked corn-based finishing diet

Item	Control	Packet^1^	SEM^2^	*P*-value
Initial BW, kg	234	233	3.9	0.37
Final unshrunk BW, kg	628	628	12.1	0.97
Adj. FSBW, kg (4%)^3^	598	608	12.8	0.53
ADG, kg				
Days 0 to 30	1.31	1.38	0.078	0.45
Days 0 to 60	1.74	1.61	0.064	0.10
Days 61 to 173	1.93	2.09	0.053	0.04
Days 173 to 203	1.16	1.21	0.09	0.74
Carc. Adj. 173-233	0.68	0.7	0.084	0.83
Adj. 0-233 (4%)^3^	1.56	1.61	0.049	0.47
DMI, kg/d				
Days 0 to 30	4.31	3.75	0.136	< 0.01
Days 0 to 60	5.5	5.44	0.172	0.71
Days 61 to 173	8.36	9.34	0.211	< 0.01
Days 173 to 203	8.56	8.99	0.217	0.15
Days 173 to 233	8.58	8.62	0.207	0.88
Days 0 to 233	7.68	8.16	0.171	0.02
Gain:Feed				
Days 0 to 30	0.305	0.371	0.0176	0.01
Days 0 to 60	0.317	0.301	0.0106	0.26
Days 61 to 173	0.233	0.225	0.0063	0.35
Days 173 to 203	0.136	0.134	0.0105	0.89
Carc. Adj. 173-233	0.076	0.08	0.0094	0.76
Adj. 0-233 (4%)^3^	0.204	0.198	0.0052	0.44

^1^The nutritional packet was formulated to provide 1.7 × 10^10^ CFU/steer-daily of *Saccharomyces cerevisiae,* 162 mg/steer-daily of vitamin C; 400 mg/steer-daily of vitamin B1; 2.4 g/steer-daily of sodium chloride, 2.4 g/steer-daily of potassium chloride, and offered during the initial and final 60 d on feed.

^2^Standard error of the mean (*N *= 30 steers per treatment).

^3^Carcass adjusted final body weight calculated as HCW divided by overall dressing percent, then multiplied by a common shrink (4%).

The carcass dressing percent of steers offered the nutritional packet was greater compared to control counterparts (62% vs. 61.1%; *P* = 0.02; Table 3). Other carcass variables, such as HCW (average = 387 kg), 12^th^ rib fat thickness (average = 18.09 mm), longissimus muscle area (average = 90.45 cm^2^), marbling score (average = 453 points), calculated yield grade (average = 3.54), quality grades (choice average = 70.04%; select average = 26.97%), and liver abscess score (average = 12.33) were unaffected by treatments (*P* ≥ 0.29).

**Table 3.** Effects of a nutritional packet on carcass characteristics and carcass quality of calf-fed steers offered a steam-flaked corn-based finishing diet

Item	Control	Packet^1^	SEM^2^	*P*-value
Hot carcass weight, kg	384	390	8.2	0.53
Dressing percent^3^	61.07	62	0.312	0.02
12^th^ rib fat, cm	18.22	17.95	1.08	0.85
LM area, cm^2^	90.33	90.56	2.022	0.93
Marbling score^4^	444	462	14.7	0.33
Calculated yield grade	3.53	3.54	0.163	0.96
Quality grade (%)				
Prime	–	–	–	–
Choice	68.51	71.56	9.728	0.79
Select	31.49	28.44	9.728	0.79
Liver abscess, %	7.41	17.24	5.133	0.29

^1^The nutritional packet was formulated to provide 1.7 × 10^10^ CFU/steer-daily of *Saccharomyces cerevisiae,* 162 mg/steer-daily of vitamin C; 400 mg/steer-daily of vitamin B1; 2.4 g/steer-daily of sodium chloride, 2.4 g/steer-daily of Potassium chloride, and offered during the initial and final 60 d on feed.

^2^Standard error of the mean (*N *= 30 steers per treatment).

^3^Dressing percent calculated as HCW divided by unshrunk final BW.

^4^300 = slight; 400 = small; 500 = modest; 600 = moderate; 700 = slightly abundant.

Regardless of treatment, a decreased rumination (*P* = 0.03) and a tendency (*P* = 0.06) for a decreased chewing activity were observed for phase 2 compared to phase 1 (Table 4). There was also a tendency for time spent resting to decrease (*P* = 0.08) during phase 2 compared to phase 1. Because the ear tag accelerometer behavior analysis included 18 steers only, the MCC data was a more robust measurement as it included 56 steers. There were multiple treatment × phase interactions observed in the MCC behavior analysis. Steers consuming the nutritional packet spent 13% less time eating during phase 1 only (*P* < 0.01) but were not different in phase 2. Steers consuming the nutritional packet had a 14% increase in meal frequency (*P* = 0.02) and a 12.3% reduction in mean meal size, kg of DM (*P* < 0.01) in phase 2, but these variables were not different in phase 1. Steers consuming the nutritional packet also had a reduced mean meal duration in both phase 1 (19.5 vs. 16.8; *P* = 0.01) and phase 2 (15.4 vs. 14.0 min; *P* = 0.01).

**Table 4.** Effects of a nutritional packet on feeding behavior of calf-fed steers offered a steam-flaked corn-based finishing diet

Item	Phase 1		Phase 2		SEM^2^	*P*-value		
	Control	Packet^1^	Control	Packet^1^		Treat	Phase	TRT × Phase
*Accelerometer (total N = 18)*								
Rumination, min/d	282.1	237.6	218.3	210.6	23.18	0.17	0.03	0.35
Eating, min/d	38.1	38.3	31.6	42.5	6.06	0.29	0.82	0.27
Chewing^3^, min/d	319.2	304.4	249.7	236.8	35.21	0.58	0.06	0.98
Resting, min/d	578.6	570.4	505.1	563.2	66.11	0.37	0.08	0.15
Active, min/d	529.9	568.9	557.7	559.1	34.33	0.53	0.71	0.44
*Meal criterion calculation (total N = 56)*								
Eating, min/d	154.0^a^	133.9^b^	79.4^c^	87.4^c^	4.58	0.16	<0.01	<0.01
Meal frequency, n/d	8.4^a^	8.4^a^	5.5^c^	6.4^b^	0.24	0.11	<0.01	0.02
Meal duration, min	19.5	16.8	15.4	14.0	0.73	0.01	<0.01	0.10
Meal size, kg DM	0.72^c^	0.70^c^	1.63^a^	1.43^b^	0.049	0.05	<0.01	0.01

^1^The nutritional packet was formulated to provide 1.7 × 10^10^ CFU/steer-daily of *Saccharomyces cerevisiae,* 162 mg/steer-daily of vitamin C; 400 mg/steer-daily of vitamin B1; 2.4 g/steer-daily of Sodium chloride, 2.4 g/steer-daily of Potassium chloride, and offered during the initial (phase 1) and final (phase 2) 60 d on feed.

^2^Standard error of the mean (*N* = 9 steers per treatment for ear tag accelerometer; *N* = 29 packet and *Nn* = 27 for control for Meal Criterion Calculation).

^3^Chewing activity calculated be adding total rumination and eating time.

No treatment × phase interactions (*P* ≥ 0.57) were observed for the digestibility variables (Table 5). The apparent digestibility of DM, OM, NDF, ADF, and hemicellulose when steers were offered the nutritional packet increased (*P* < 0.01) by 2.45%, 2.55%, 8.59%, 10.65%, and 7.64% respectively.

**Table 5.** Effects of a nutritional packet on apparent total tract nutrient digestibility of calf-fed steers offered a steam-flaked corn-based finishing diet

Item	Phase 1		Phase 2		SEM^2^	*P*-value		
	Control	Packet^1^	Control	Packet^1^		Treat	Phase	TRT × Phase
*Intake during digestibility assessment, kg/d*								
Dry Matter	6.81	6.37	7.83	7.87	0.78	0.61	< 0.01	0.41
Organic Matter	6.45	6.03	7.31	7.35	0.74	0.60	< 0.01	0.40
NDF	1.23	1.16	1.40	1.42	0.14	0.67	< 0.01	0.40
ADF	0.44	0.42	0.46	0.47	0.05	0.88	0.05	0.43
Hemicellulose	0.79	0.73	0.94	0.95	0.09	0.56	< 0.01	0.39
*Total Tract Nutrient Digestibility, %*								
Dry Matter	84.62	86.75	82.19	84.21	0.57	< 0.01	< 0.01	0.89
Organic Matter	85.46	87.84	87.16	89.30	0.63	< 0.01	< 0.01	0.78
NDF	61.71	67.30	68.85	75.56	1.83	< 0.01	< 0.01	0.68
ADF	59.76	66.32	63.77	71.90	1.89	< 0.01	< 0.01	0.57
Hemicellulose	62.80	67.86	71.34	77.39	1.86	< 0.01	< 0.01	0.72

^1^The nutritional packet was formulated to provide 1.7 × 10^10^ CFU/steer-daily of *Saccharomyces cerevisiae,* 162 mg/steer-daily of vitamin C; 400 mg/steer-daily of vitamin B1; 2.4 g/steer-daily of sodium chloride, 2.4 g/steer-daily of potassium chloride, and offered during the initial and final 60 d on feed.

^2^Standard error of the mean (*N* = 30 steers per treatment).

Steers consuming the nutritional packet had an increased (*P* = 0.02) average papillae area of approximately 30% compared to the control (0.08 vs. 0.11 cm^2^), which was only numerically reflected on other ruminal morphological indicators of absorption (Table 6). Average papillae number was unaffected by treatment (*P* = 0.39). The ruminal fluid content of steers offered the nutritional packet tended (*P* = 0.09) to have an 8% greater total ruminal VFA (mM) concentration, while individual molar proportions of acetate, butyrate, propionate, valerate, isobutyrate, isovalerate, branched chain VFA, and as well as C2:C3 ratio were not affected (*P* ≥ 0.48) by treatments.

**Table 6.** Effects of a nutritional packet on ruminal characteristics of calf-fed steers offered a steam-flaked corn-based finishing diet

Item	Control	Packet^1^	SEM^2^	*P*-value
*Volatile fatty acid profile*				
Total VFA, mM	87.90	95.85	3.28	0.09
Acetate^3^	41.32	41.09	0.98	0.86
Propionate^3^	35.02	34.22	0.85	0.51
C2:C3 Ratio	1.20	1.25	0.06	0.56
Butyrate^3^	10.01	10.33	0.06	0.68
Valerate^3^	4.29	4.35	0.35	0.89
Isobutyrate^3^	7.66	8.15	1.16	0.77
Isovalerate^3^	1.68	1.86	0.18	0.48
Branched chain VFA^3^	9.34	10.01	1.08	0.66
*Papillae morphology*				
Average papillae area, cm^2^	0.08	0.11	0.01	0.02
Absorptive surface area, cm^2^/fragment-cm^2^	11.70	13.96	1.10	0.15
Average papillae number *n*/cm^2^	69.59	64.65	4.30	0.39
Papillae absorptive area, %	88.89	91.57	1.25	0.14

^1^The nutritional packet was formulated to provide 1.7 × 10^10^ CFU/steer-daily of *Saccharomyces cerevisiae,* 162 mg/steer-daily of vitamin C; 400 mg/steer-daily of vitamin B1; 2.4 g/steer-daily of sodium chloride, 2.4 g/steer-daily of potassium chloride, and offered during the initial and final 60 d on feed.

^2^Standard error of the mean (*N *= 30 steers per treatment).

^3^Measured as: mM/ 100 mM of tVFA.

## DISCUSSION

The objective of this study was to evaluate the effects of a nutritional packet containing live yeast (*S. cerevisiae*), vitamin C (ascorbic acid), vitamin B1 (thiamin), and the electrolytes NaCl and KCl on growth performance, carcass characteristics, nutrient digestibility, feeding behavior, papillae morphology, and ruminal VFA profile of steers in a calf-fed system consuming a steam-flaked corn-based finishing diet. The 6% greater intake seen throughout the entire feeding period for the steers offered the packet aligns with a meta-analysis performed by Desnoyers et al. (2009) who reported an overall increased DMI by 1.2% (34.6 vs. 35.0 g/kg of BW) when *S. cerevisiae* was supplemented to dairy cows. Magrin et al. (2018) also reported a 5.5% DMI increase for live yeast supplemented bulls being finished on a high concentrate diet. An overall DMI increase of 9% was also reported by Finck et al. (2014) when supplementing live yeast to newly weaned and received crossbred steers.

The increased ADG for packet-supplemented steers during the intermittent period, where the nutritional packet was not offered, indicates a carry-over effect of the packet ingredients. Such an effect was also reported by Theurer et al. (2019) when offering high-risk heifers *S. cerevisiae boulardii* CNCM I-1079 at a rate of 1 g/steer-daily (20 × 10^9^ CFU/steer-daily) only during the first 45 d receiving period. They reported an ADG increase of 4% and a 5% improvement in feed efficiency for the entire 232 d of the finishing phase.

Steers consuming the nutritional packet in the current experiment were more efficient when consuming the packet during the first 30 d post feedlot arrival, a period characterized by dietary changes (adaptation to grain) and considered as one of the most stressful moments for calf-feds. This positive outcome is consistent with the findings of Zinn et al. (1999) who evaluated the effects of a yeast culture on transit stressed beef calves in the feedlot-receiving phase. The greater feed efficiency can be attributed to several factors acting together, which stems from the strategic combination of the nutritional packet ingredients. Schaefer et al. (1997) reported the benefits of electrolyte supplementation of a dissolvable solution of Na, K, and Mg prior to and following transit. A reduction in live weight loss and the normalization of factors such as blood lactate levels in transported cattle were reported by Schaefer et al. (1992); hence, perhaps similar beneficial effects occurred during post transit supplementation in the current study. Because the first 30 d post feedlot arrival (receiving phase) is a high-stress period, the consumption of electrolytes may have aided in restoring proper acid-base and water balance and by preventing excessive intracellular and extracellular fluid loss (Coffey et al., 2001), allowing the animal to return to homeostasis at a faster rate during this time of high stress. The live yeast and vitamin B1 likely acted together to create a more stable ruminal environment through balancing lactate-producing and utilizing bacteria. Wang et al. (2014) reported beneficial ruminal microbiome responses (a reduction in *Streptococcus bovis* and an increase in *Megasphaera elsdenii*) and subsequent increase on ruminal pH average through thiamin supplementation in dairy cows offered a subacute ruminal acidosis (**SARA**) inducing diet with high inclusions of corn grain. Ogunade et al. (2019) reported similar beneficial rumen responses with *S. cerevisiae* supplementation to Holstein steers offered a 50% concentrate diet. The greater efficiency observed in the receiving phase may also be supported by the findings of Ogunade et al. (2019), where live yeast enhanced cellulolytic bacteria fermentation when offered to step-up diets (diets containing greater fiber content compared to finisher diets). This corroborates with the greater digestibility of the fiber components NDF, ADF, and hemicellulose for steers consuming the nutritional packet in the present experiment.

The anti-oxidant effects of vitamin C offer protection against oxidative stress and damages caused by free radicals, thus, improving immune responses (Frei et al., 1989; Washko et al., 1993; Wolf, 1993; Matsui, 2012). Moreover, live yeast supplementation improves animal health by reducing bovine respiratory disease as reported by Theurer et al. (2019). They supplemented *S. cerevisiae* for the initial 45 d post feedlot arrival to high-risk heifers stepped up to a steam-flaked corn-based finishing diet. The nutraceutical properties of the nutritional packet may be further investigated to address potential animal health improvements.

When cattle are offered high-concentrate diets inducing SARA, thiamin concentrations in the rumen and blood decreased, which induced an increase in pro-inflammatory cytokine hematic concentrations (Pan et al., 2016). Pan et al. (2017) reported that hematic and ruminal concentrations of thiamin were reduced when animals were offered a diet containing ground corn at a 35% inclusion (DM basis). A thiamin-deficient rumen can cause pyruvate to accumulate, which in turn will be likely converted to lactate by the lactate dehydrogenase (Wang et al., 2014; Kumar et al., 2015), which may act synergistically with other acidosis-causative factors. An acidotic environment impedes absorption of thiamin through the rumen epithelium (Pan et al., 2017). Furthermore, a thiamin-deficiency poses a polioencephalomalacia (PEM) risk, indicating a relationship between acidosis and PEM (Brent, 1976). Pan et al. (2017) presented evidence suggesting that thiamin supplementation can counteract these negative factors and stated that thiamin supplementation can alleviate inflammation through the reduction of pro-inflammatory cytokines. Moreover, it has been shown that an increased concentration of dietary thiamin through strategic supplementation (180 mg/kg DMI to Holstein cows offered a 33.2% starch diet) supports a positive ruminal microbial growth, which leads to a more efficient fermentation (Pan et al., 2017). The latter researchers reported that thiamin supplementation stimulates cellulolytic and lactate utilizing bacteria growth, thus supporting an improved ruminal fermentation and increased ruminal pH. Thiamin derivatives such as thiamin pyrophosphate (TPP) are essential for several biochemical pathways like the pentose phosphate pathway, which is responsible for the metabolism of five-carbon sugars. These simple sugars are necessary for nucleotide production in order to favor the multiplication of ruminal micro-organisms; therefore, thiamin supplementation supports a healthy microbiome in the rumen (Wang et al., 2014). The bacterial species supported by thiamin include lactate-utilizing bacteria, thus reducing the risk of lactic acidosis. Damage of ruminal papillae cells caused by low ruminal pH can be reduced by thiamin in goats offered a corn grain-based diet, as reported by Zhang et al. (2020). Such research findings are in agreement with the improvement in ruminal papillae morphology observed in the present experiment.

Both digestibility assessments conducted (phases 1 and 2) resulted in similar positive outcomes for steers consuming the nutritional packet. Along with the increased digestibility, a tendency for an increase in total VFA concentration was observed in the rumen at the time of slaughter. The greater digestion of nutrients paired with the increased total VFA concentration can be accredited to the nutritional packet components causing positive effects within the rumen. Live yeast has been reported to increase oxygen-scavenging within the rumen, which allows obligate anaerobic micro-organisms to flourish and increase fermentation and digestion (Newbold et al., 1996). Additionally, yeast supports cellulolytic bacteria growth, and both live yeast and thiamin supports lactate cross-feeding bacteria (McAllister et al., 2011). This is perhaps the reason why steers in the current experiment had an improved digestibility of nutrients in both phases and exhibited a greater concentration of total VFAs in the rumen. However, since rumen fluid was only taken at harvesting time, this is only speculation, as VFA differences were not enough to trigger effects in feed efficiency during phase 2.

The improved ruminal papillae area observed concurs with the improved ruminal wall health observed by Ma et al. (2021) and Garcia-Diaz et al. (2018), who reported a reduced papillae stratum corneum thickness when *S. cerevisiae* was supplemented to goats offered a whole corn-based diet. Healthier and more efficient ruminal papillae could perhaps be attributed to a less acidic ruminal pH as observed by Pan et al. (2016). In the latter study, a rumen pH increase occurred when thiamin was supplemented to Holstein dairy cows offered a SARA inducing diet with 35% ground corn inclusion (DM basis). Additionally, Ogunade et al. (2019) reported an improvement in ruminal pH through live yeast supplementation when Holstein steers were offered a 50% concentrate ration.

The combination of greater digestibility, increased VFA concentration, and a larger capacity for nutrient absorption through an improved ruminal wall, could be the driving force behind the advantage in the early gain performance and in the final dressing percentage. In this instance, greater available energy is diverted towards the synthesis of useable carcass tissues since maintenance requirements were exceeded.

Cattle rumination is regulated by particle size and forage NDF level in a diet (Allen, 1997) and because steers were undergoing step-up diets for half of the initial 60 d, it was expected to observe an increased rumination when animals were being offered diets with higher inclusions of fiber. In addition, because rumination is a variable included in the chewing time calculation, a similar effect was observed for the behavior trait of chewing. In general, steers consuming the packet had smaller meals and a greater meal frequency, which is beneficial to the animal since they did not overload their digestive tract. This potentially allowed the rumen to increase fermentation and digestion of ingested nutrients, which is supported by the superior digestibility in both phases and the increased total VFA concentration observed. Through the phenomenon of a higher frequency of smaller meals, the risk of creating acidotic ruminal conditions may be reduced (Bach et al., 2007). These researchers also observed a greater rumen pH in the *S. cerevisiae* supplemented group of dairy cows, which also had an increased meal frequency.

Acidotic conditions can damage rumen papillae as reported by Ma et al. (2021) and Zhang et al. (2020). Although pH was not analyzed in the current study, it can be hypothesized that due to the smaller, more frequent meals of the packet supplemented steers, these animals developed a healthier rumen, as indicated by the increased papillae area. The effects of other companion ingredients of the nutritional packet ingredients such as vitamin C, vitamin B1, and electrolytes on feeding behavior have yet to be explained.

## CONCLUSION

The nutritional packet offered to calf-fed steers improved feed efficiency during the initial 30 d after feedlot arrival, induced overall superior feed intake, nutrient digestibility, and smaller-more frequent meals that seemed to last until cattle harvest. Packet supplementation improved dressing percentage, ruminal papillae area, and tended to release more total ruminal VFA concentration. Regardless of treatment, rumination and chewing activities diminished towards the end of the finishing phase of calf-fed steers offered a steam-flaked corn-based diet. Current evidence suggests the need for continued research investigating optimal combinations of key nutrients and probiotic supplements that promote digestive tract health.
